# Primary Retinal Cultures as a Tool for Modeling Diabetic Retinopathy: An Overview

**DOI:** 10.1155/2015/364924

**Published:** 2015-01-19

**Authors:** Andrea Matteucci, Monica Varano, Cinzia Mallozzi, Lucia Gaddini, Marika Villa, Sara Gabrielli, Giuseppe Formisano, Flavia Pricci, Fiorella Malchiodi-Albedi

**Affiliations:** ^1^Department of Cell Biology and Neuroscience, Istituto Superiore di Sanità, Viale Regina Elena, 299, 00161 Rome, Italy; ^2^GB Bietti Eye Foundation IRCCS, Via Livenza, 3, 00198 Rome, Italy; ^3^Department of Technology and Health, Istituto Superiore di Sanità, Viale Regina Elena, 299, 00161 Rome, Italy

## Abstract

Experimental models of diabetic retinopathy (DR) have had a crucial role in the comprehension of the pathophysiology of the disease and the identification of new therapeutic strategies. Most of these studies have been conducted *in vivo*, in animal models. However, a significant contribution has also been provided by studies on retinal cultures, especially regarding the effects of the potentially toxic components of the diabetic *milieu* on retinal cell homeostasis, the characterization of the mechanisms on the basis of retinal damage, and the identification of potentially protective molecules. In this review, we highlight the contribution given by primary retinal cultures to the study of DR, focusing on early neuroglial impairment. We also speculate on possible themes into which studies based on retinal cell cultures could provide deeper insight.

## 1. Introduction

Diabetic retinopathy (DR) is the leading cause of blindness in working-age population in developed countries. Despite the recent introduction of antivascular endothelial growth factor (VEGF) therapies against vision-threatening diabetic macular edema, which has remarkably improved its prognosis, DR is still one of the most disabling sequelae of diabetes and a heavy socioeconomic burden.

In the symptomatic phase of the DR, key clinical features are alterations of the vascular system. These abnormalities initially are represented by vessel leakage, due to increased microvascular permeability, and microaneurysm formation. In the progression of the disease, endothelial and pericyte cell death and formation of acellular capillaries intervene, decreasing the blood supply. Consequences are ischemia and stimulated growth of fragile and leaky blood vessels, leading eventually to severe vision loss. For the dramatic sequelae of blood vessel damage, DR has been considered for a long time exclusively a microangiopathy. It is now evident, however, that the neuroglial components of the retina are affected before any retinal vasculature involvement. This consideration stems from the observation that deficits of the neural retina may be detected even in total absence of retinal microvessel damage. Increasing evidence suggests that functional alterations, such as the impairment of color vision [1, 2], loss of contrast sensitivity [3], alterations in the electroretinogram [4–6], and thinning of retinal layers evaluated by optical coherence tomography [7], can be evidenced in patients before DR is diagnosed by the detection of microangiopathy during ophthalmoscopic examination, supporting a direct damaging effect of the diabetic* milieu* on the neuronal population. While it cannot be excluded that damage to the vasculature may have already started at a microscopic level before observable signs of leakage or hemorrhage, data on the well-known neurotoxic effects of hyperglycemia (see, for a review, [8]) strongly supports the hypothesis that retinal neuronal damage may precede microangiopathy. Furthermore, focusing exclusively on angiopathy may have caused an underestimation of the role of the functional interaction existing between blood vessels and the neuronal component. The crosstalk, where Müller cells play a crucial link, may have possible consequences on retina pathology. While it is easy to perceive how microvasculature damage can affect neurons in conditions such as ischemia and hemorrhage, it is more difficult to demonstrate if and how neurons can influence microvasculature function. However, the simple observation that changes in the exposure to light modify blood flow highlights how the two compartments are closely linked together and can be functionally coupled [9] and suggests that neuronal dysfunction may influence blood vessel physiology. A better knowledge of the process of neuroglial involvement during the early phases of diabetes could therefore help further characterize the physiopathology of the disease and, more importantly, envision possible precocious therapeutic strategies involving neuroprotection.

*In vitro* studies have substantially contributed to the characterization of the pathophysiology of retinal damage during diabetes (see for a review [10]). Cell culture models provide simplified systems where the effects of different potentially toxic factors present in the diabetic* milieu*, such as hyperglycemia, glutamate, and advanced glycation end products (AGEs), can be investigated in an isolated context. While studies on endothelial and pericyte cell cultures have amply contributed to highlighting diabetes-triggered mechanisms of cell damage (see, for example, [11–14]), the studies on retina neuroglial cultures have been more limited, although a wide literature exists on the neurotoxic effects of high glucose (HG) concentrations (see for a review [8]).

In this review, we illustrate how dissociated and organotypic primary retinal cultures have been successfully applied in the study of DR, focusing on early neuroglial damage induced by the diabetic* milieu*.

## 2. Neurodegeneration as an Early Event during Diabetic Damage to the Retina: The Relevance of Neuroprotection

In experimental models of DR, there is a prominent loss of retinal cells* in vivo* [15–18]. An analogous cell loss is observed* in vitro*, following exposition of cell cultures to HG [19–21]. Considering the prominence of vascular damage in the symptomatic phase of the disease, most interest has been focused on endothelial and pericyte cell death. However, convincing evidence also points to retinal neurons as object of cell loss [17, 22, 23].

Data from experiments performed on cell cultures have remarkably contributed to highlight the direct toxic effects of exposure to HG conditions on the neuronal population (see for a review [8, 10]). Neurotoxicity has been investigated in several types of CNS primary neuronal cell cultures, such as dorsal root ganglion cells (DRGC) [24], cortical [25] and hippocampal neurons [26], and retinal ganglion cells [21, 27]. These models are particularly relevant to highlight the early events in DR. HG-treated cultures, in fact, can mimic the condition solely due to diabetic glucose concentration, in absence of the events correlated to microvascular abnormalities. The experimental procedures to mimic hyperglycemia used glucose concentrations ranging from 25 mM to 50 mM. It could be argued that 50 mM glucose is way beyond what would ever occur* in vivo*, even in pathological conditions. It should be noted, at this regard, that some primary cultures, such as DRGCs, require basal 25 mM glucose to grow [24, 28] and a decrease to 5 mM glucose, corresponding to normal glycemia, leads to increased DRGC neuronal death. In these cell models, to produce a hyperglycemic insult, a 20 mM additional glucose was required. However, in other experimental models, including retinal neurons [26, 29–31], a glucose concentration not exceeding 30 mM was sufficient to induce neuronal cell death. In some cases, HG-induced increase in apoptosis was reported to be caspase independent [32]. Other studies have, in contrast, described caspase-dependent mechanisms [27, 33, 34]. A point that still needs to be clarified is which type of neuronal cells is more sensitive to the damaging effect of HG. Generally ganglion cells are considered as a principal target, both* in vivo* [17, 35–37] and* in vitro* [27, 38–40]. In addition, amacrine [41] and photoreceptor [42, 43] cell death has been observed in animal models of DR. To our knowledge, HG-induced, cell type-specific susceptibility has not been addressed by the use of primary cell cultures.

In both* in vivo* and* in vitro* models, a number of growth factors have been described as crucial in retinal neurons survival in the course of diabetes [44–47]. Changes in the pathways activated by neurotrophic factors, which modulate growth, differentiation, and survival of neurons, have been described as potential pathogenetic mechanisms in DR. Nerve growth factor (NGF), the first discovered neurotrophic factor, is deeply implicated in DR pathogenesis [36, 44, 48] and studies on cell cultures have contributed to the characterization of its mechanism of action [49, 50]. In diabetes, an impaired production of matrix metalloproteinase-7, which cleaves the NGF precursor proNGF, has been observed, leading to the decrease in NGF and to the accumulation of proNGF, which binds to p75NTR and preferentially activates a proapoptotic pathway [49]. In RGCs, a cell line of immortalized retinal ganglion cells that express NGF and its receptors, TrkA and p75NTR, diabetes-induced peroxynitrite formation has been shown to impair TrkA receptor phosphorylation and activate the p75NTR-dependent proapoptotic pathway, leading to neuronal cell death [50]. Consistently, silencing of p75NTR protected against HG- and proNGF-induced apoptosis in RGC cultures [51]. Recently, the ganglion cell origin of immortalized RGC line, also known as RGC-5, has been denied since photoreceptor markers were detected [52]. Therefore, conclusions obtained using these cells that relate to retinal ganglion cell-specific responses should be reconsidered, using primary retinal neuronal cultures. Brain-derived neurotrophic factor (BDNF) and neurotrophin 4 (NT-4) are other members of the neurotrophic factor family, with important roles in development, differentiation, and maintenance of neurons [53, 54]. In retinal tissue cultures, BDNF and NT-4 significantly reduced HG-induced retinal ganglion cell apoptosis and increased the number of regenerating neurites [21, 27]. Moreover, BDNF has been shown to protect retinal cell cultures from hyperglycemia via the TrkB/ERK/MAPK pathway [20].

VEGF and pigment epithelial growth factor (PEDF) have been object of increasing interest because of their ability to stimulate or inhibit, respectively, retinal angiogenesis. Considering its role in retinal neovascularization, VEGF has been thoroughly studied as a possible target to contrast retinal vascular pathology in DR, on the basis of the dramatic improvement obtained with anti-VEGF drugs in the therapy of age-related macular degeneration, another serious vasculopathy of the retina. On the basis of these results, ranibizumab, a monoclonal antibody fragment against VEGF, is now prescribed in the therapy of diabetic macular edema. Both VEGF and PEDF, besides their role in modulating blood vessel growth, show neurotrophic and neuroprotective abilities. These properties, however, have not been the object of comparable interest. Studies on* in vivo* and* in vitro* DR models, which focused on possible modulation of neuronal homeostasis by both factors, would be of great interest, especially considering that long-term anti-VEGF therapy, contrasting a neurotrophic factor, might endanger a neuronal population that is already compromised due to the chronic exposure to the diabetic* milieu*.

Studies using retinal cell cultures have been applied to investigate the activity of neuroprotective factors. Apart from growth factors, data have been obtained on the neuroprotective activity of citicoline, taurine-conjugated ursodeoxycholic acid (TUDCA), and erythropoietin [21, 55, 56].

In conclusion,* in vitro* retinal models have been profitably applied to study HG-induced retinal neurodegeneration, focusing on glucose neurotoxicity and on the role of growth factors, such as NGF and BDNF, in neuroprotection.

## 3. Müller Glia Involvement in DR

Müller cells, together with astrocytes, belong to retinal macroglial cells. While astrocytes have a very restricted localization, being confined to ganglion cell and nerve fiber layers, Müller cells span the entire retinal thickness, sending projections to both ganglion cell layer, where they terminate with expanded endfeet at the inner border of the retina, and photoreceptor layer, where they extend into the space surrounding the photoreceptor segments. Overall, they are responsible for a series of crucial functions, such as glutamate buffering, blood-retinal barrier maintenance, control of osmotic and ionic balance, and modulation of neuronal activity, all aimed at maintaining the homeostasis of the retinal microenvironment [57, 58].

Müller glia is deeply involved in DR, showing morphological and functional alterations from the early phases of the disease. Overexpression of glial fibrillary acidic protein (GFAP), a sign of Müller glia activation, is one of the first histologic findings in DR-affected retina [59–62]. Later on, impairment of functional activities of Müller glia intervenes, including downregulation of inwardly rectifying K^+^ currents [63, 64], decreased activity of L-glutamate/L-aspartate transporter (GLAST) [65] and impaired ability to convert glutamate to glutamine [66]. Moreover, Müller glia is considered to contribute to DR inflammatory scenario [67–71]. These considerations have suggested that Müller glia activation contributes to the degeneration of neural retina. Other results indicate, on the contrary, that Müller glial activation is not inevitably dangerous [72] and may be even neuroprotective [73–75], at least in the early phases of the disease [30]. The issue is highly debated [76, 77].

Primary Müller glia cultures or immortalized Müller glia cell lines have been frequently used in the investigations aimed at unraveling the significance of Müller glia involvement in DR.

Several data have been obtained* in vitro* on the role of Müller glia in inflammation. Exposure to HG of MIO-M1 cells, an established Müller cell line, induced increased production of Receptor for AGEs (RAGE), which, via MAPK pathway, activates cytokine production. Blockade of RAGE prevented cytokine responses [78]. The production of nitric oxide (NO) and prostaglandin E(2), mediators of inflammation, and the expression of iNOS and COX-2 were found to be increased in rMC-1 Müller cell line upon exposure to HG [79]. Müller cells are among the principal source of both VEGF and PEDF, which have proinflammatory and antiinflammatory activities, respectively [80–83]. In cultured retinal Müller cells under HG conditions, levels of VEGF increased, while those of PEDF decreased, thus inducing a proinflammatory and proangiogenic phenotype in Müller glia [84]. In addition, PEDF contrasted HG-induced decrease in glutamate uptake and in Kir4.1 and GLAST expression [85].

Another debated issue is Müller cell death in DR. Data on Müller cell loss are mainly based on* in vitro* experiments, where Müller cells are supposed to die following exposure to HG conditions (25–30 mM glucose) [86–89]. These results are, however, not univocal. Most of data supporting Müller cell death are derived from immortalized Müller cell lines or cultivated human Müller cells. Data from rat primary Müller cell cultures are controversial. In some studies, no cell death was observed after 72 h [30] or after 5 days in HG [90], while others detected Müller cell apoptosis after 72 h in the same conditions, possibly related to AKT inhibition [33]. In favor of an apoptotic mechanism is the observation that in rMC-1 and in cultured human primary Müller cells incubated in HG conditions, glyceraldehyde-3-phosphate dehydrogenase (GAPDH) translocates to the nucleus [91]. A similar nuclear accumulation of GAPDH in Müller cells was observed in retinal sections from diabetic rats at 4 months of diabetes [91].

To complicate even more the topic, it has been recently proposed that Müller cell loss may be due to pyroptosis, a type of cell death recently described, which depends on the activation of the caspase-1/interleukin- (IL-) 1*β* pathways (for a review, see [92]). It has been proposed that this type of cell death may involve Müller cells [18] since caspase-1/interleukin- (IL-) 1*β* pathways are activated* in vitro* in Müller cells following exposure to HG [93, 94]. Furthermore, IL-1*β* can significantly increase GAPDH nuclear accumulation in Müller cells [94]. This cell death type is, however, difficult to identify, since it is not accompanied by characteristic morphological nuclear stigmata, such as apoptosis or necrosis, but it is associated with hyperplastic change, according to the authors who described it [18]. Further studies are required to assess to which extent Müller cells pyroptosis intervenes in the pathophysiology of DR.

In summary, the studies on primary Müller cells or established Müller cell lines, such as MIO-M1 or rMC-1, have contributed to highlight the role of Müller cells in the inflammatory scenario in diabetes.

## 4. Mechanisms of Diabetic* Milieu*-Mediated Injury to Neural Retina

*In vitro* studies have substantially contributed to the characterization of the pathophysiology of neuroglial damage induced by the potentially neurotoxic compounds present in the diabetic* milieu*. Most mechanisms are initiated by hyperglycemia, which affects the cells of all tissues in diabetes. Its detrimental effect is especially evident in the insulin-independent tissues, in which glucose uptake into the cells does not require the action of insulin and leads to a high intracellular concentration of the sugar. In the past decades, several studies have demonstrated that the pathogenetic cascade of diabetes-induced complications is associated with the metabolic consequences of excess glucose disposal, either through repeated acute changes in cellular glucose metabolism or through the long-term accumulation of altered products. Collected evidence has elucidated several mechanisms through which the diabetic* milieu* causes harmful consequences, often pointing to a wide interconnection of different pathways [95–109]. In this review we will focus on the diabetes-induced metabolic impairments that have been described as mostly involved in the neurodegenerative process that occurs in DR: (i) oxidative stress and its correlated presence of NO, peroxynitrite, and AGEs; (ii) excitotoxicity; (iii) inflammation.

Oxidative stress has been the object of considerable interest as mediator of retinal injury. DR is characterized by an increased level of oxidative/nitrosative stress in the retinal tissue [110–112]. The production of NO and superoxide anion is elevated, while the antioxidant enzymes are downregulated and glycated, a modification that limits their capacity to detoxify oxygen radicals [112, 113].* In vitro*, coculture experiments, where mice retinal neurons were co-cultivated with Müller cells, demonstrated that large release of NO can induce neuronal cell death [114]. In addition, NO may react with superoxide anion, leading to the formation of highly toxic peroxynitrite, which is noxious to endothelial cells [115] and RGCs [50] and activates Müller glia [51]. One major source of cellular redox species is dysfunctional mitochondria. An inhibitor of mitochondrial electron transport chain complex II has been found to normalize superoxide production in HG-treated rMC-1, suggesting that mitochondria were primarily responsible for increase in superoxide production. Several data on the efficacy of antioxidants, such as TUDCA, an endogenous bile acid [116], or aminoguanidine, an inhibitor of the inducible isoform of NO synthase [117], have been obtained* in vitro*.

Another potential source of oxidative stress is related to the presence of AGEs. Chronic exposure to HG leads to the formation of glycated biomolecules and the progressive accumulation of AGEs through the increased chemical (nonenzymatic) reaction of reducing sugars with amino groups [118, 119]. The initial glycation is followed by a cascade of chemical reactions resulting in the formation of intermediate products (Schiff base, Amadori and Maillard products) and finally to a variety of derivatives, named AGEs. These products are extremely reactive and can interact with circulating or tissue proteins, altering their functions and structures. In addition, they may interact with specific receptors, such as RAGE [119, 120]. Finally, this pathway has been demonstrated to lead to oxidative stress and trigger proinflammatory signaling, implicated in endothelial dysfunction, arterial stiffening, and microvascular complications [121–123]. Studies* in vitro* have analyzed the effects of AGEs on the retinal neuronal population, evidencing a depressing influence on the neuritic regeneration [124] and a stimulated proapoptotic trend [34, 125–127]. In addition, they showed AGEs effects on Müller glia. In retinal explants, AGEs induced an increase in GFAP immunolabeling [34] and in MIO-M1 cells, and blockade of RAGE prevented cytokine responses induced by HG and S100B [78]. Using a coculture system, AGEs significantly increased the expression of MCP-1 in retinal neurons, which activated microglial cells, increased microglial migration, and upregulated secretion of TNF-*α* [128].

Oxidative stress is also among the consequences of excitotoxicity. In this condition, an excessive stimulation of glutamate receptors results in uncontrolled intracellular calcium flow in neurons and cell death. Reactive oxygen and nitrogen species are produced as a consequence of activation of calcium-dependent enzymes, such as phospholipase A2 and nitric oxide synthase and by dysfunctional mitochondria [129]. Besides DR, excitotoxicity is involved in other neurodegenerative diseases of the retina, including glaucoma and ischemia [130–132]. Numerous studies indicate a disruption of glutamate homeostasis in DR [133]. Human diabetic patients have an elevated vitreal glutamate concentration [134] and increased glutamate levels have been reported in the retinas of diabetic rats [60, 135], probably due to an impairment of glutamate metabolism [136]. Interestingly, it has been shown in isolated Müller cells that oxidative damage accounted for both the decreased activity of GLAST and the reduction of the cell specific enzyme glutamine synthetase. Downregulation of this enzyme, which converts glutamate to glutamine, can contribute to glutamate-induced excitotoxicity, thus creating a self-perpetuating vicious circle between oxidative stress and excitotoxicity. Both organotypic and dissociated retinal cultures have been used as* in vitro* experimental models of retinal excitotoxicity, addressing both the study of mechanisms [137–140] and the identification of neuroprotective agents [85, 141–144].

The involvement of both chemical mediators and inflammatory cells in DR supports a pathogenetic role of inflammation in the disease [145]. Elevated levels of proinflammatory cytokines, such as IL-1*β*, IL-6, and IL-8, tumor necrosis factor-*α* (TNF-*α*), and vascular cell adhesion molecule-1, have been found in the vitreous in patients with proliferative DR [146–148]. Increased VEGF and IL-6 levels have been detected in the aqueous humor of diabetic patients with macular edema [149]. Microglia activation is considered a histologic hallmark of DR and appears early in the course of the disease, before the onset of overt neuronal cell death [150]. Although the role of inflammation in vascular changes has been well established and indirect evidence links inflammation with vascular apoptosis, a precise causal relation between inflammation and neuronal apoptosis has not yet been defined [22]. Possible mechanisms involve microglial activation [150] and cytokine-activated neurodegenerative pathways [151–153]. Another point deserving further investigation is microglia-Müller glia crosstalk, which has recently been described as a critical mechanism in the modulation of retinal response to injury [154].

**Figure 1:**
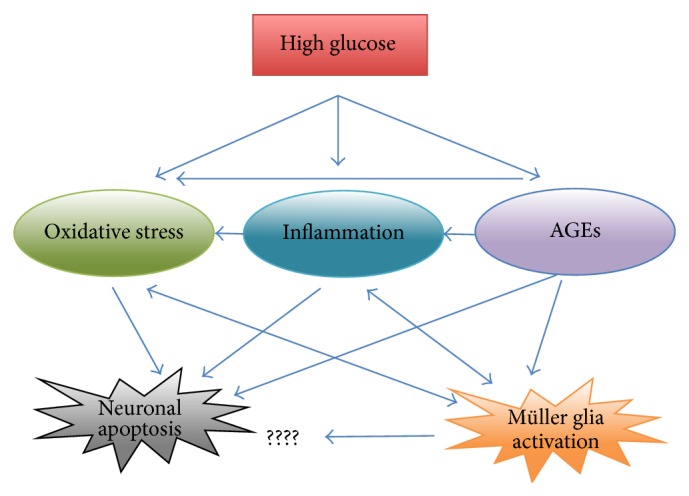
The diagram summarizes the various interacting pathways leading to oxidative stress upon exposure to high glucose concentrations. Most of the pathways are considered to play a role in retinal neuron apoptosis and Müller glia activation. It is still controversial if Müller glia activation is also responsible for neuronal cell death.

In conclusion, the results of investigations from retinal cell cultures have confirmed how the different pathogenetic pathways leading to HG-induced cell damage (oxidative stress, inflammation, formation of AGEs) are amply interconnected, as summarized in Figure 1.

## 5. Primary Retinal Cultures

*In vitro* systems allow the study of retinal cells with strict control of environmental factors, providing excellent experimental model to investigate pathogenetic mechanisms, test pharmacological compounds, and examine in depth findings emerging from* in vivo* studies [155]. For several decades, experiments have been performed on immortalized cell lines, which can be expanded* in vitro* with no limitations. However, immortalized cell lines are usually derived from tumor cells and have often lost the original tissue specificity and phenotype. It is of particular importance for assuring reliable and useful results when using immortalized cell lines to guarantee cell authenticity, since it is estimated that 15% to 20% of the cells used in experiments are not what they are supposed to be [156]. Primary cell cultures, on the contrary, more closely mimic* in vivo* tissues and are therefore preferred to model physiopathologic conditions, despite their demanding technical requirements.

There are some fundamental problems that impact on the predictive value of test systems based on cell cultures. Living organisms maintain a tightly controlled, stable environment that is fundamental for cellular physiology; on the contrary,* in vitro* experimental models undergo dramatic changes due to depletion of nutrients, reduced availability of oxygen, and accumulation of waste products. As a simplification of the complex* in vivo* situation, cell cultures usually include few or only one cell type growing in an artificial environment and the lack of interaction between cells and their physiological environment is another critical point. In addition, the differentiation state and the response patterns of the cells in culture are different, with respect to the* in vivo* conditions [157]. Finally one of the most frequently cited limitations of* in vitro* tests is qualitative and quantitative deficiencies in the biotransformation of chemicals used in experimental models [158]. Nevertheless the broad use of* in vitro* tests in academic and industrial research in recent years confirms that they are still considered a useful tool to investigate cellular physiology and pathological conditions of many diseases.

In the study of neuronal and glial components of the retina, two types of primary cell cultures have been more frequently used: dissociated retinal cultures, where retinal cells are enzymatically and/or mechanically dispersed before seeding, and organotypic cultures, where the entire retina or retinal fragments are dissected and cultivated as intact tissue. More recently, impressive progress has been made in the field of retinal cell cultures due to the development of stem cell technology. Induced pluripotent stem cells, in particular, have opened new and exciting scenarios for their outstanding potential in reconstructing retinal architecture, which envision possible future advancements in retinal transplant therapy. This type of cultures in which excellent reviews have been recently published [58, 159–162] will not be considered in the present paper.

In the field of DR, both dissociated and organotypic retinal cultures have been used and have contributed to gain further insight especially into the early effects induced by HG on the neuronal and glial compartments of the retina.

### 5.1. Dissociated Cultures

**Figure 2:**
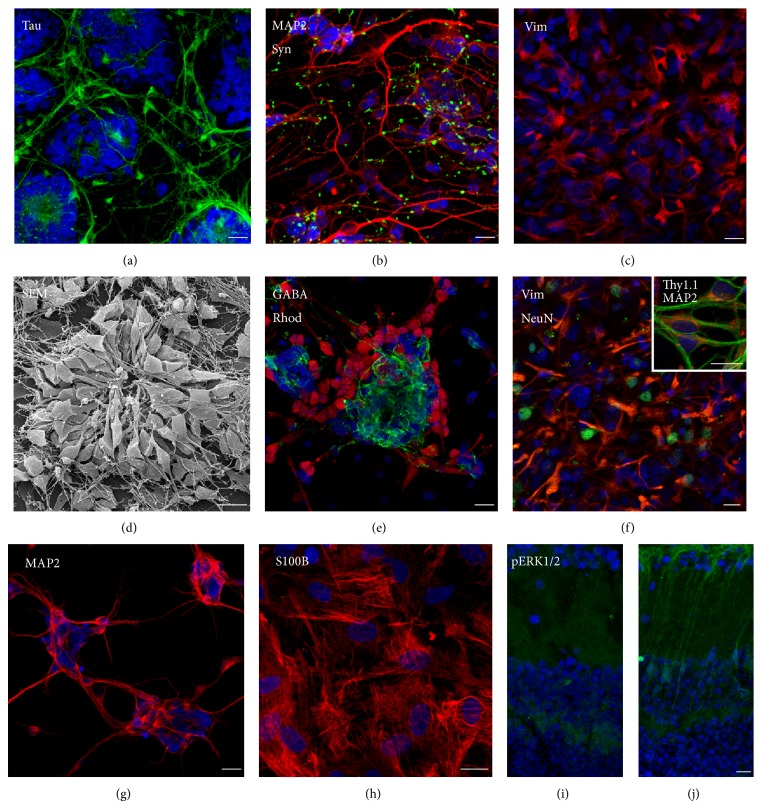
Morphologic and immunocytological characterization of dissociated ((a)–(h)) and organotypic ((i), (j)) primary rat retinal cultures. In dissociated mixed cultures, the heterogeneous cell population is composed of differentiated neurons, labeled for axonal (Tau, (a)) or dendritic (MAP2, (b)) microtubule-associated proteins and synaptophysin (Syn) (b) and of Müller cells, positive for vimentin (Vim) (c). In the neuronal population, photoreceptors form rosette-like aggregations (SEM, (d)), positive for rhodopsin (e). The positivity for the inhibitory neurotransmitter GABA indicates the presence of amacrine or horizontal cells (e). Thy1.1-immunolabeled ganglion cells are also present ((f), inset). Coimmunostaining of vimentin, a marker of Müller cell cytoskeleton, and NeuN, which labels neuronal nuclei, proves the mixed neuronal/glial composition of the cell culture (f). Following special culturing protocols, retinal cell cultures can be enriched in neurons (g) or in Müller cells, as shown by the specific marker S100B (h). In organotypic cultures, retinal tissue structure in preserved. In (j), Müller glia projections, positive for pERK1/2 after short-term treatment of HG, cross the inner plexiform layer and expand in the ganglion cell layer. (i) Control sections. Bar = 10 *μ*m.

**Figure 3:**
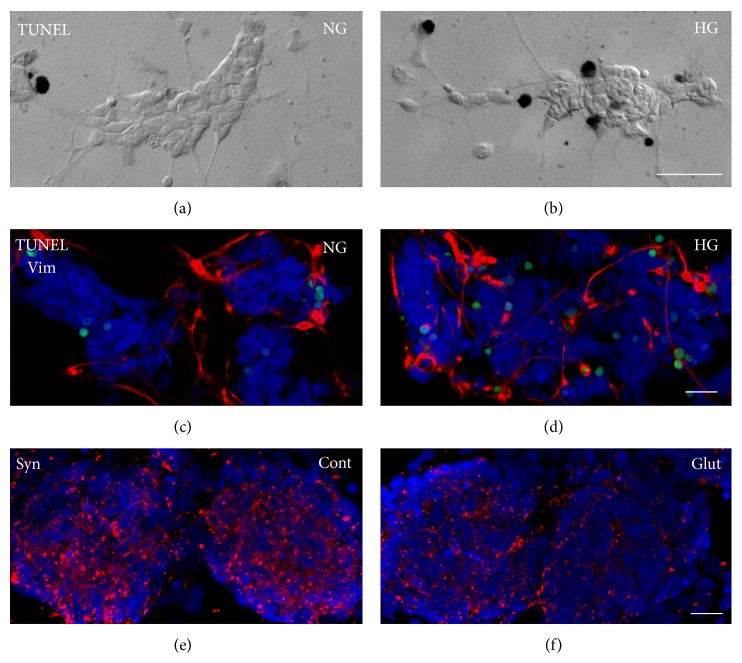
Retinal cultures can be used to investigate the direct effects of various pathological conditions in retinal cells. In (b), colorimetric TUNEL assay highlights an increase in apoptotic nuclei in HG-treated pure neuronal cultures, with respect to control cultures (a). In HG-treated mixed cultures, the same treatment (d) induces an enhanced reactivity for vimentin (red), suggesting an activation of glial cells, with respect to normal glucose cultures (c). In (d), TUNEL-positive apoptotic nuclei are labeled in green. In (f), the reduction of synaptophysin (Syn) immunoreactivity in glutamate treated cultures, with respect to control cultures (e), suggests a damage of synaptic structure, confirming the excitotoxic effect of excessive glutamate concentration. Bar = 20 *μ*m.

Several techniques have been described to obtain dissociated primary retinal cultures [155]. In our protocol [143, 163], retinas are obtained from embryos or pups at early postnatal days; after dissection, they are incubated for 15 min at 37°C in a balanced salt solution containing enzymes able to dissociate the tissue. The enzymes are then removed and finally inactivated with cell culture media containing 10% of fetal calf serum. After the enzymatic dissociation, retinas are triturated through polished glass pipettes of decreasing tip size to produce a suspension of dissociated cells. Retinal cells are finally seeded in cell culture plates or on coverslips previously coated with poly-L-lysine to favor cell adhesion. In these conditions, retinal cells grow as a mixed cell population, composed of both glial and neuronal cells. They tend to assume a complex organization, with some structures reminiscent of the retina* in situ* (Figures 2(a)–2(h)). Müller glia forms a monolayer, which after 10 and 20 days* in vitro* (DIV) can become confluent. Neurons grow on top of the glial layer and differentiate. The heterogeneous cell population is composed of differentiated neurons, labeled for axonal (Tau, (a)) or dendritic (MAP2, red, (b)) microtubule-associated proteins and synaptophysin (Syn, green, (b)) and of Müller cells, positive for vimentin (c). In the neuronal population, photoreceptors form rosette-like aggregations (Scanning Electron Microscopy, (d)), positive for rhodopsin (green, (e)). The positivity for the inhibitory neurotransmitter GABA indicates the presence of amacrine or horizontal cells (red, (e)). Thy1.1-immunolabeled ganglion cells (orange) are also present ((f), inset). Coimmunostaining of vimentin (red), a marker of Müller cell cytoskeleton, and NeuN (green), which labels neuronal nuclei, proves the mixed neuronal/glial composition of the cell culture (f). Following special culturing protocols, retinal cell cultures can be enriched in neurons, positive for MAP2 (g), or in Müller cells, as shown by the specific marker S100B (h). These types of retinal cultures can be used to investigate neuroglial damage, such as that induced by HG or glutamate exposure (Figure 3).

In ophthalmologic research, dissociated retinal cultures have been used to identify factors able to promote cell survival and/or differentiation [143, 164–173] and evaluation of artificial vitreous substitute biocompatibility (see for a review [163]).

In the studies on DR mechanisms, the* in vitro* approach has given the opportunity to investigate the direct effects of the potentially toxic compounds present in the diabetic* milieu*, excluding the effects mediated by vascular changes and allowing a simulation of the early stages of the disease. In mixed retinal cultures, Costa et al. [31] demonstrated that TNF-*α* is responsible for HG-induced cell death and that blocking the activity of its receptor is an adequate strategy to avoid cell loss under the influence of the diabetic hyperglycemia. Notable evidence suggests that glutamate may be involved in retinal neurodegeneration in DR, as discussed above, and retinal cell cultures have been useful to gain further insight into the role of excitotoxicity. In cultured retinal cells, elevated concentrations of glucose induce changes in the protein levels of several ionotropic glutamate receptor subunits and alter the Ca^2+^ homeostasis of retinal neurons [32]; moreover, HG treatment increases the evoked release of D-aspartate in the retina [174]. Elevated glucose concentration induces early changes in AMPA receptors in cultured retinal neurons, whereas the same receptors do not appear to be affected by elevated glucose concentration in cultured hippocampal neurons. Thus, retinal AMPA receptors seem to be more susceptible to regulation by elevated glucose concentration than hippocampal AMPA receptors [175]. HG conditions also modify the adenosinergic system in cultured retinal neurons, whose modulation is evidenced by the altered expression of the receptors A_1_AR, A_2_AR, and A_3_AR [176]. It has been repeatedly suggested that oxidative stress may play a role in DR, although it is not clear yet whether it has a primary role in the pathophysiology of the diabetic complications or it is simply a consequence of them [177]. In order to protect retinal neurons against oxidative stress in DR, various antioxidant compounds, such as TUDCA and erythropoietin, have been tested in primary retinal cultures [116, 169].

Following special culturing protocols, retinal cell cultures can be enriched in Müller cells or neurons, giving rise to virtually pure glial or neuronal cell cultures. To obtain pure Müller cells cultures, mixed cultures are passaged in order to eliminate neuronal cells [30]. In other protocols, mixed cultures are used after a few weeks from seeding, when all neurons have died and the cultures contain only Müller cells [178]. Aggregates containing neuronal cells and cellular debris may also be removed by vigorously washing mixed cell cultures with medium at 6-7 DIV. The procedure yields a purified Müller cell preparation, which can be maintained for several weeks [179, 180]. Müller cells are increased in number and activated in both DR patients and experimental models [181] and primary cultures of Müller cells have been profitably used to study the HG-induced alterations in this cell type. Chronically elevated glucose causes a significant decrease in AKT activity in cultured Müller cells and the observed downregulation of this survival signaling pathway can be responsible at least in part for HG-induced apoptosis [33]. Müller cells produce various neuroprotective factors [76] and Müller cell cultures have been used to investigate the role of these factors in early DR. Zhu et al. [182] demonstrated, that under HG conditions, glial-derived neurotrophic factor may play important role in protecting Müller cells during the early stages of DR. In primary Müller cell cultures, Jiang et al. [183] described the protective effect of melatonin under hyperglycemic conditions; interestingly, melatonin attenuated HG-induced VEGF overproduction. HG stimulation of Müller cells increased VEGF secretion through a mechanism involving ERK1/2. Targeting ERK1/2 signaling pathway with U0126, an ERK1/2 specific inhibitor, able to reduce VEGF secretion in Müller cells, could be investigated as anti-VEGF treatment for DR [184].

Pure neuronal cultures can be obtained changing the medium of dissociated mixed cultures after a few hours from seeding, thus removing most of glial cells, which tend to adhere more slowly than neurons. In addition, at DIV 1, 5 *μ*M arabinosyl-cytosine is added to prevent the proliferation of residual glial cells. Pure neuronal cultures have not been widely used in experimental models of DR. Recently, by comparing HG effects in mixed neuronal/glial and pure neuronal retinal cultures, we observed that the presence of Müller cells, in the early phases, protects retinal neurons against the damaging effects of HG [30].

### 5.2. Organotypic Cultures

Organotypic cultures (also referred to as retinal explants or retinal tissue cultures) allow preserving the architecture of the tissue* in situ*, maintaining the advantages of easy manipulation and treatment of* in vitro* models. Organotypic cultures of the retina can be maintained* in vitro* without the need of tissue slicing, as required for other central nervous system tissues, thereby providing a better preservation of retinal tissue cytoarchitecture and most intraretinal connections (Figures 2(i) and 2(j)). In contrast, when the eye bulb is separated from the optic nerve, due to optic nerve sectioning, the connection between the eye and the brain is lost [185], leading to a physiological thinning of the ganglion cell layer [186, 187]. This experimental model, including neurons, glial cells, and a relevant component of vasculature, can provide information resulting from the complex crosstalk between the retinal cell types. In addition, it allows analyzing the effects of the potentially toxic compounds present in the diabetic* milieu* on the whole retina in absence of the confounding metabolic homeostasis induced by systemic diabetes, as in* in vivo* models. Moreover, maintaining mature neurons* in situ*, it represents an excellent tool for studying neurodegenerative events.

To set up retinal explant cultures, animals are euthanized and eye bulbs are immediately dissected and transferred to a Petri dish containing ice-cold sterile buffer. Eyes are then incised, cornea and lens are removed, and the whole retina is carefully dissected from the sclera. Several methods have been developed to maintain retinal explants* in vitro*, involving the usage of different substrates on which retinal tissues are placed for support [188–191]. More recently, Thangaraj et al. [187] have described an explant culture system, without any substrate, able to better evaluate the development of photoreceptors and outer segments* in vitro*. The general organization of the retina and the maintenance of extracellular matrix are well preserved in retinal explants for long culture periods; moreover, the distribution of retinal layers, the maturation of the retina, and the location of most cell types are similar to those observed in the retina* in situ* [192–196]. Recently, a method to set up human organotypic retinal cell cultures has been developed [197].

This cell culture model has been widely used to investigate the direct effects of HG on retinal cells. For example, in retinal explants the expression of HO-1 is upregulated in response to HG; the persistence of hyperglycemic insult reduces the expression of HO-1, suggesting that long-term hyperglycemia leads to an increase in reactive oxygen species generation and decreased antioxidant capacity [198]. Oshitari and Roy [199] demonstrated in retinal explants that HG accelerates retinal neuronal cell death and that the pathological changes involve Bax-mediated retinal neuronal cell death. The same authors described a neuroprotective and regenerative effect of BDNF, NT-4, and citicoline against HG-induced apoptosis [27]. The effects of AGEs have also been investigated in this cell model. Lecleire-Collet et al. [34] showed in retinal explants that AGEs induce retinal neurodegeneration and that cells in the ganglion cell layer appear to be the most sensitive. The neurotoxicity of AGEs in retinal tissue is linked to increased expression of AIF and caspase-9. In retinal explants, NT-4 has a neuroprotective and regenerative role correlated with the reduction of caspase-9 and AIF expression [124].

### 5.3. *In Vitro* Studies: Future Perspectives

Unsettled issues still exist regarding glial and neuronal engagement in DR and experiments based on primary cell cultures can give an answer at least to some of them. The observation that impairment of color vision and contrast sensitivity arises very early in diabetes suggests that photoreceptor function may be precociously affected by the diabetic* milieu*. Some recent work supports this observation. Photoreceptors were found to be a major source of superoxide in mice diabetic retina [200] and degenerative change in photoreceptors was observed before neuronal cell type damage [201]. In addition, in Ins2Akita mouse, photoreceptor cell death preceded that of amacrine and ganglion cells [202]. However, a precise time course of HG-induced neuronal cell damage has not been fully characterized. Studies on primary cell cultures, where the different neuronal cell types can be identified by means of immunocytochemical techniques (Figures 2 and 3) can allow monitoring HG damage in retinal cells, evidencing possible different susceptibility. Another interesting issue, still unsettled, is the role of Müller glia, that is, whether protective or detrimental. In a recent study, we observed that in mixed cell cultures short-term HG treatment did not induce neuronal apoptosis, while activated Müller glia showed increase in phosphorylated ERK1/2 (pERK1/2) levels, which trigger a neuroprotective pathway. In long-term HG treatment, in contrast, neuronal apoptosis increased, while pERK1/2 signaling was inactive in Müller glia [30]. These results suggest that the role of Müller glia may change depending on the different conditions and emphasize how* in vitro* models can be usefully applied in this context. Finally, the possibility of setting up cocultures can provide valuable information on the interactions between different cell components under the influence of the diabetic* milieu*, for example, on Müller glia and microglia crosstalk [154, 203, 204].

## 6. Conclusions

In this review, we illustrate how dissociated and organotypic primary retinal cultures have been successfully applied in the study of DR, focusing on early neuroglial damage induced by the diabetic* milieu*.

It is generally agreed that tight control of glycemia can reduce the risk of DR and control its progression. Once the disease has developed, many treatments are available for its clinical management, using several approaches. Laser photocoagulation represents the standard of care for newly diagnosed DR. Intravitreal drugs, such as corticosteroids and recently introduced anti-VEGF, are alternative options, the latter prescribed in diabetic macular edema. Vitrectomy and vitreoretinal surgery to remove scarring and hemorrhage is normally reserved for advanced complications of proliferative DR. Notwithstanding these varieties of approaches, DR still represents a clinical challenge. The increasing evidence pointing to early neuroglial involvement in DR, preceding microangiopathy, suggests that further knowledge on the mechanisms at the basis of this involvement could help clarify if innovative therapeutic strategies including neuroprotection could be beneficial to contrast disease progression. The results highlighted in this review suggest that primary retinal cultures may contribute to shed further light on the mechanisms leading to HG-induced neurodegeneration and identifying neuroprotective pathways.
